# On-chip inter-modal Brillouin scattering

**DOI:** 10.1038/ncomms15819

**Published:** 2017-07-07

**Authors:** Eric A. Kittlaus, Nils T. Otterstrom, Peter T. Rakich

**Affiliations:** 1 Department of Applied Physics, Yale University, New Haven, Connecticut 06520, USA

## Abstract

Brillouin nonlinearities—which result from coupling between photons and acoustic phonons—are exceedingly weak in conventional nanophotonic silicon waveguides. Only recently have Brillouin interactions been transformed into the strongest and most tailorable nonlinear interactions in silicon using a new class of optomechanical waveguides that control both light and sound. In this paper, we use a multi-mode optomechanical waveguide to create stimulated Brillouin scattering between light-fields guided in distinct spatial modes of an integrated waveguide for the first time. This interaction, termed stimulated inter-modal Brillouin scattering, decouples Stokes and anti-Stokes processes to enable single-sideband amplification and dynamics that permit near-unity power conversion. Using integrated mode multiplexers to address separate optical modes, we show that circulators and narrowband filters are not necessary to separate pump and signal waves. We also demonstrate net optical amplification and Brillouin energy transfer as the basis for flexible on-chip light sources, amplifiers, nonreciprocal devices and signal-processing technologies.

## Abstract (web summary)

Here, Kittlaus *et al*. demonstrate stimulated inter-modal Brillouin scattering on-chip. Through this process, a Brillouin interaction couples light fields that propagate in distinct spatial modes of a Brillouin-active silicon waveguide, which may allow a variety of new processes in silicon photonics.

Stimulated Brillouin scattering (SBS) is an optomechanical three-wave interaction that produces coherent coupling between optical waves and acoustic phonons. Within waveguides, Brillouin interactions are remarkably tailorable, permitting a range of hybrid photonic–phononic signal processing operations including tunable narrowband filtering^1^,^2^,^3^, nonreciprocal processes^4^,^5^,^6^,^7^ and frequency synthesis^8^,^9^ that have no all-optical analogues. Strong Brillouin interactions have only recently been created in silicon using a new class of optomechanical waveguides^10^,^11^,^12^,^13^ that produce record-high nonlinearities and admit intriguing dynamics^2^,^3^,^8^,^11^,^13^ within a variety of new travelling-wave optomechanical systems^2^,^3^,^8^,^14^. Further control of Brillouin processes in silicon waveguides could enable phenomena such as mode cooling and nonreciprocal transparency^7^,^15^,^16^,^17^, and lead to new lasers, oscillators, filters^1^,^8^,^9^,^10^,^11^,^12^,^13^,^14^,^15^,^16^,^17^,^18^,^19^,^20^,^21^ and signal-processing technologies^4^,^22^,^23^,^24^,^25^ in silicon photonics.

Many different types of Brillouin interactions are possible within microscale waveguides and devices, each producing a distinct set of phenomena. To date, strong forward-SBS (FSBS), also termed stimulated Raman-like scattering^8^, has been achieved within silicon waveguides^10^,^11^,^12^,^13^. Through FSBS, phonons mediate coupling between co-propagating light fields that are guided in the same optical mode. This interaction produces very strong optical nonlinearities (∼10^4^ times larger than in silica fibres)^10^,^11^, enabling large net amplification^13^ and cascaded energy transfer. This interaction also produces dynamics that are very different than those of the widely studied backward-SBS (BSBS) interaction^26^, offering intriguing opportunities for new processes and phenomena. For example, FSBS is uniquely suited to hybrid photonic–phononic signal-processing schemes based on phonon emit/receive operations that have recently been realised in silicon^2^. However, unlike BSBS, FSBS does not produce single-sideband gain. As a result, it is nontrivial in many cases to adapt BSBS device concepts and established techniques for signal processing^22^,^23^,^24^, slow light^27^ and filtering^1^,^18^,^19^,^20^,^21^ using FSBS interactions.

Alternatively, within multi-mode optomechanical waveguides it is also possible to create phonon-mediated coupling between light fields guided in distinct spatial modes. This process, termed stimulated inter-modal Brillouin scattering (SIMS), effectively decouples the Stokes and anti-Stokes processes, enabling single-sideband gain and powerful nonlinear dynamics that differ from those of conventional forward- and backward-SBS processes. For example, through FSBS both Stokes and anti-Stokes scattering processes are mediated by the same phonon mode. As a result, the FSBS process yields symmetric scattering of light to many successive blue- and red-shifted orders, fundamentally limiting the magnitude of energy transfer^8^,^13^. By comparison, the phase-matching requirements of SIMS produce a form of dispersive symmetry breaking that causes the Stokes and anti-Stokes processes to couple to different phonon modes. As a result, the dynamics of these processes become decoupled, permitting single-sideband amplification as well as a variety of processes unique to inter-modal scattering^5^,^28^.

Stimulated inter-modal Brillouin scattering and SIMS-like processes have previously been demonstrated in multi-mode and microstructured fibres.^28^,^29^,^30^,^31^, admitting new physics compatible with both established technologies based on BSBS as well as many novel optical devices. Fibre experiments have used SIMS processes to create single-sideband amplification^28^, self oscillation^31^ and active optical isolation^5^, with dynamics that permit near-unity energy transfer between pump and signal waves^28^,^32^,^33^. Inter-modal scattering in microsphere resonators permits attractive schemes for optical cooling^15^, and for Brillouin-scattering-induced transparency^7^,^17^, an acousto-optic analogue of electromagnetically induced transparency that permits highly efficient slow-light generation and nonreciprocal energy storage. All of these processes, and many others^1^,^4^,^6^,^9^,^18^,^19^,^20^,^21^,^22^,^23^,^24^ become available on a silicon chip if engineerable forms of SIMS can be created.

In this paper, we demonstrate stimulated inter-modal Brillouin scattering on-chip for the first time. Through this process, a Brillouin interaction couples light fields that propagate in distinct spatial modes of a Brillouin-active silicon waveguide. This system decouples Stokes and anti-Stokes processes through symmetry breaking based on multi-mode dispersion. Harnessing this interaction, we demonstrate single-sideband optical amplification and unidirectional Brillouin energy transfer in silicon. Combining this form of Brillouin coupling with on-chip spatial mode multiplexers offers a powerful new approach to Brillouin-based signal processing in silicon. Strong Brillouin coupling enables 3.5 dB of single-sideband small-signal gain, corresponding to 2.3 dB of net on-chip amplification in this low propagation loss system. Pump and signal waves are coupled in and out of separate optical modes of a single Brillouin-active silicon waveguide using integrated mode multiplexers. This enables independent on-chip control of pump and signal waves without additional optical components such as isolators or narrowband filters. At higher guided-wave powers, this same device produces 50% stimulated inter-modal energy transfer between these two fields. The realization of highly engineerable SIMS in silicon unlocks new strategies for adapting and controlling Brillouin interactions in silicon photonic systems.

## Results

### Operation scheme

**Figure 1:**
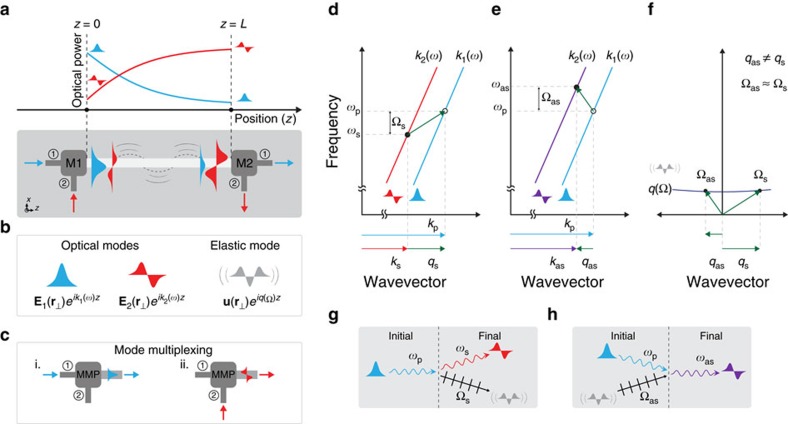
On-chip inter-modal Brillouin scattering. (**a**) Operation scheme. Pump and Stokes waves are coupled into the fundamental (symmetric) and first-excited (anti-symmetric) modes of a waveguide through separate ports of an integrated mode multiplexer. While passing through the active device region, energy is transferred from pump to Stokes. At the end of the device, the two waves are demultiplexed through an identical mode multiplexer. (**b**) Schematic of the three modes participating in the Brillouin process. Two optical modes—one even and the other odd in electric field symmetry—are coupled through the interaction with an elastic mode with an even displacement profile. (**c**) Diagram of two-port mode multiplexer operation: Light injected into port 1 is coupled into the symmetric mode of a waveguide, whereas light coupled through port 2 is coupled into the same waveguide’s anti-symmetric mode. (**d**–**f**) Diagrams showing dispersion relations for the participating modes. (**d**) depicts phase matching and energy conservation for a Stokes process, whereas (**e**) shows an anti-Stokes process. (**f**) plots the dispersion relation for the Brillouin-active acoustic mode. Note that phonons mediating Stokes and anti-Stokes processes have wavevector different in magnitude and sign, but nearly identical frequencies. (**g**,**h**) depict Feynman-like scattering diagrams for Stokes and anti-Stokes processes, respectively, which show the initial and final particle states of the system.

We explore stimulated inter-modal energy transfer and single-sideband amplification using the multi-port optomechanical system diagrammed in Fig. 1a. This system consists of a multi-mode hybrid photonic-phononic waveguide that is interfaced with two integrated mode multiplexers labelled M1 and M2.

We consider the interaction between two guided optical modes 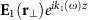 and 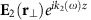 and a Brillouin-active acoustic phonon mode 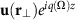, as sketched in Fig. 1b. Here 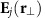 and *k*_*j*_(*ω*) are the electric field profile and wavevector of the *j*^th^ optical mode at frequency *ω*, and 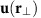 and *q*(Ω) are the elastic displacement field profile and wavevector of the acoustic mode at frequency Ω.

Each port of the optical mode multiplexers, denoted as M1 and M2 in Fig. 1c, maps to a distinct spatial mode as shown in Fig. 1b. In the absence of nonlinear coupling, light injected into port 1 of M1 propagates in the symmetric mode (**E**_1_) of the optomechanical waveguide, exiting the system in port 1 of M2. Similarly, light entering port 2 of M1 couples to the anti-symmetric mode (**E**_2_) of the waveguide, exiting the system in port 2 of M2. However, when pump and signal waves are injected into ports 1 and 2, respectively, resonant nonlinear coupling to the Brillouin-active phonon mode (**u**) scatters energy from the symmetric mode (**E**_1_) to the anti-symmetric mode (**E**_2_), producing both active mode conversion and single-sideband signal amplification.

This stimulated scattering process is driven by optical forces within the optomechanical waveguide segment. The pump wave (**E**_1_) interferes with the signal wave (**E**_2_) to produce a time-modulated optical force that excites a travelling-wave phonon (**u**), which in turn scatters energy from pump to signal. This process, which is a type of SIMS, produces amplification and mode conversion through a stimulated Stokes process. In contrast to FSBS processes that have previously been demonstrated in silicon waveguides^10^,^11^,^12^,^13^, this process produces single-sideband amplification and unique forward scattering wave dynamics as a basis for new types of nonreciprocal devices^5^,^7^,^17^.

Single-sideband amplification is possible in this system because the Stokes and anti-Stokes processes are no longer mediated by the same phonon mode. This is understood from the distinct phase-matching conditions for these processes. Through the Stokes process, diagrammed in Fig. 1g, a pump photon (*ω*_p_, *k*_1_(*ω*_p_)) guided in the symmetric mode (**E**_1_) scatters to a red-shifted photon (*ω*_s_, *k*_2_(*ω*_s_)) guided in the anti-symmetric mode (**E**_2_), and a guided Stokes phonon (Ω_s_, *q*_s_). For this process to occur, both energy conservation (Ω_s_=*ω*_p_−*ω*_s_) and phase matching (*q*_s_=*k*(*ω*_p_)−*k*(*ω*_s_)) must be satisfied. Combined, these conditions require *q*_s_=*k*_1_(*ω*_p_)−*k*_2_(*ω*_p_−Ω_s_). We use this condition as the basis for a succinct diagrammatic representation that includes both phase matching and energy conservation, as seen in Fig. 1d. A phonon that satisfies this condition must lie along the acoustic dispersion curve *q*(Ω) sketched in Fig. 1f. This phase-matched phonon, which connects the initial (open circle) and final optical states (solid circle) sketched in Fig. 1d, is a forward-moving phonon.

Through the anti-Stokes process, diagrammed in Fig. 1h, the same pump photon (*ω*_p_, *k*_1_(*ω*_p_)) combines with a guided phonon (Ω_as_, *q*_as_) to produce a blue-shifted photon (*ω*_as_, *k*_2_(*ω*_as_)). In this case, phase matching and energy conservation require *q*_as_=*k*_1_(*ω*_p_+Ω_as_)−*k*_2_(*ω*_p_), which differs from the Stokes process. As seen from Fig. 1e, the anti-Stokes phonon that connects the initial optical state (open circle) and final optical state (solid circle) is a backward-moving phonon.

Since the Stokes and anti-Stokes phonons are distinct (that is, *q*_s_≠*q*_as_) symmetry is broken between the two processes. As a result, the dynamics for the Stokes and anti-Stokes processes are decoupled, permitting unidirectional single-sideband coupling between only two optical fields. (for further discussion see Supplementary Note 4).

This dispersive symmetry breaking significantly alters the dynamics of the stimulated Brillouin scattering process. Through conventional intra-modal FSBS processes, a phonon generated through stimulated Stokes scattering can subsequently mediate an anti-Stokes scattering process. However, through SIMS this is no longer the case, as these two processes are mediated by distinct phonon modes. As a consequence, only thermally-populated phonon modes can mediate anti-Stokes processes, whereas the Stokes process, which produces coherent phonon emission (Fig. 1g), becomes a stimulated scattering process.

### Multi-mode optomechanical waveguide

This multi-port optomechanical system is fabricated from a single-crystal silicon layer using an SOI fabrication process (for details see Methods). The spatial mode multiplexers M1 and M2 address the individual modes of the optomechanical waveguide and are fabricated on the same layer as the Brillouin-active waveguide segment. These mode multiplexers utilize asymmetric mode-selective directional couplers^34^,^35^ adapted to the low-loss ridge waveguide designs used here (see Supplementary Note 3 for details). Fibre arrays and grating couplers transfer light into single-mode waveguides, which serve as the input and output ports of M1 and M2.

**Figure 2:**
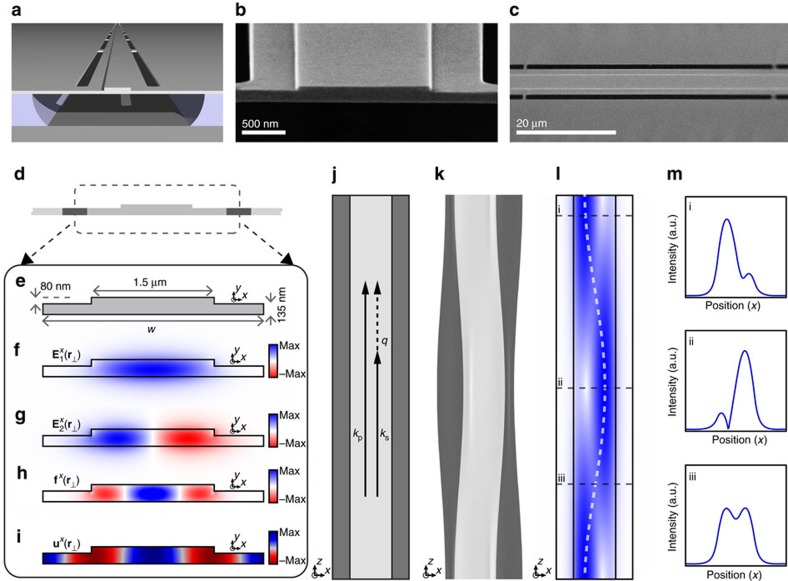
SIMS-active waveguide. (**a**) Schematic of suspended Brillouin-active waveguide (**b**) SEM of device cross-section. The scale bar represents 500 nm in length. (**c**) Top-down SEM of suspended device, with a scale bar representing 20 μm. (**d**) Diagram of device cross-section. Dashed region is plotted in (**e**) with relevant dimensions listed. (**f**,**g**) are **E**_x_ fields of the first two guided optical modes. (**h**) *x*-component of the electrostrictive force generated by these optical modes. (**i**) *x*-displacement field of the ∼6 GHz Brillouin-active acoustic mode. (**j**–**l**) sketch top-down views of a 14 μm long section of the device including phase matching (**j**), elastic displacement for the membrane and ridge regions (**k**), and intensity beating of the two propagating optical modes (**l**). Three slices of the intensity profile in **l** are plotted in **m** (i–iii).

The Brillouin-active waveguide segment that supports stimulated inter-modal coupling is a suspended ridge waveguide of the type depicted in Fig. 2a. Scanning electron micrographs (SEMs) show the cross-section (Fig. 2b) and top view (Fig. 2c) of the crystalline silicon device. The Brillouin-active waveguide is suspended over a 2.3 cm distance by an array of nanoscale tethers placed every 50 μm (Fig. 2c). The active region consists of a 80 nm × 1.5 μm wide ridge on a 135 nm thick silicon membrane of width *w*=2.85 μm. The cross-section of the active device region, highlighted in Fig. 2d, is diagrammed in Fig. 2e.

Both light and sound are guided within the membrane-suspended waveguide of Fig. 2e. Light is confined to the central ridge structure through total internal reflection, which guides the co-propagating TE-like optical modes. The symmetric (**E**_1_) and anti-symmetric (**E**_2_) spatial modes are plotted in Fig. 2f–g at a wavelength of *λ*=1,550 nm. The computed effective phase indicies for these two modes are 2.8 and 2.7, respectively. The membrane structure also supports many higher-order modes in the ridge and slab; however, we selectively excite only the symmetric (fundamental) and anti-symmetric (first-excited) modes throughout this experiment. Elastic waves are confined to this same structure due to the large acoustic impedance mismatch between silicon and air.

This system supports numerous guided elastic waves with longitudinal, shear and flexural character. Of these, only a small number are Brillouin-active and mediate transfer of energy between modes (**E**_1_) and (**E**_2_). In addition to phase matching, Brillouin coupling requires appreciable overlap between optical force density, produced by the interference between (**E**_1_) and (**E**_2_), and the elastic displacement field. One such Brillouin-active phonon mode that produces strong inter-modal Brillouin coupling at 6 GHz frequencies is shown in Fig. 2i,k. Figure 2i shows the dominant (*x*-component) of elastic displacement field (**u**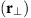) within the waveguide cross-section, and Fig. 2k is a top-view showing elastic distortion of this same mode. Comparing the electrostrictive optical force distribution of Fig. 2h, with Fig. 2i, we see that the transverse displacement produces good overlap with the optical forces to mediate coupling.

### Stimulated inter-modal scattering

**Figure 3:**
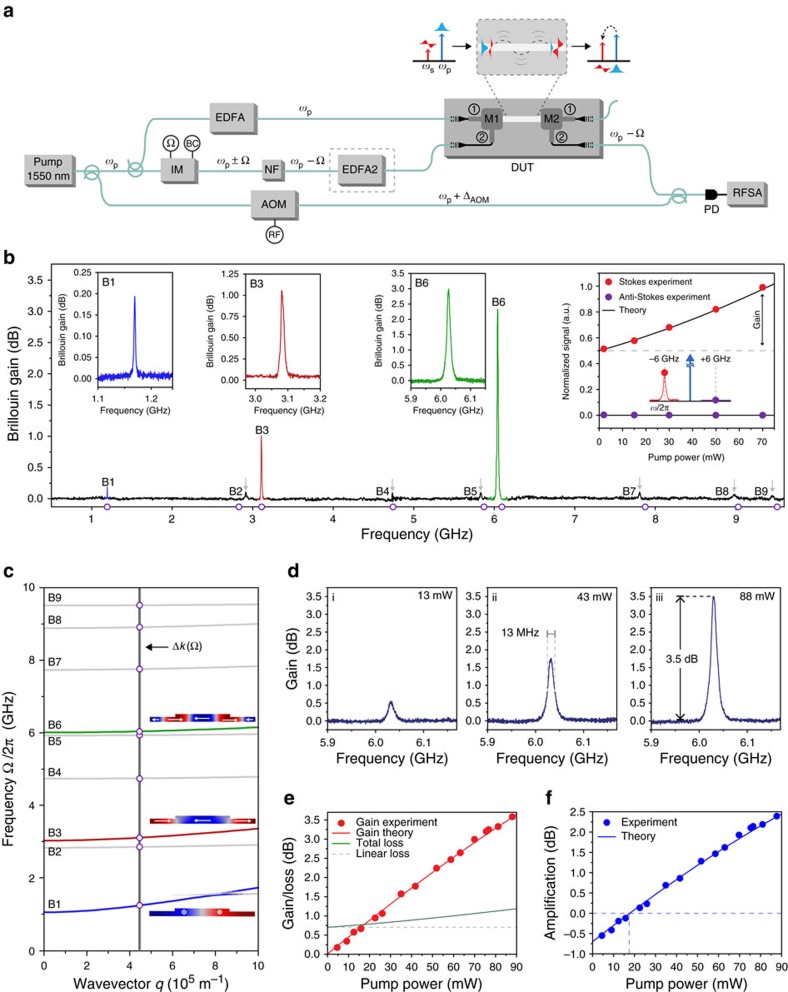
Experimental results showing on-chip SIMS and net optical amplification. (**a**) Diagram of the experimental apparatus. A laser operating around 1,550 nm is used to synthesize a strong pump wave through an EDFA while a separate branch is passed through an intensity modulator and notch filter to generate a frequency-shifted probe wave. An additional EDFA, denoted EDFA2, is inserted later for the optical energy transfer experiment. Pump and probe waves couple in and out of the device through mode multiplexers. After the device, the probe wave is combined with a frequency-shifted reference arm, and its intensity is measured via heterodyne detection. (**b**) SIMS spectra over a 9 GHz span showing several Brillouin-active acoustic modes, with three highlighted and plotted in more detail in insets. Simulated frequencies from multi-physics simulations are denoted by violet circles along the abscissa. A fourth inset shows single-sideband gain as a function of pump power when driving acoustic resonance B6—note that while Stokes light experiences gain, no light is scattered to the anti-Stokes order. A data trace at high pump power is included schematically, showing single-sideband gain as the probe wave is swept through the Brillouin resonance. Red and purple dots correspond to the measured gain when driven on-resonance. (**c**) Calculated dispersion curves for the observed Brillouin-active acoustic modes. Displacement is diagrammed for modes B1, B3 and B6 next to their respective curves. The vertical gray line plots the wavevector of Brillouin-active phonons under the conditions tested. (**d**–**f**) show Brillouin gain and amplification data for the strongest Brillouin-active mode B6. (**d**i–iii) Plot inter-modal Brillouin gain spectra obtained for three different pump powers. These data show a narrowband Brillouin resonance at 6.03 GHz. (**e**) plots peak gain (red), linear loss (dash) and total loss (green) versus on-chip pump power. (**f**) Net on-chip amplification from (**e**), obtained by subtracting total loss from optical gain. These data are measured with the pump wave in the symmetric optical mode and the probe wave in the anti-symmetric mode. IM, Mach–Zehnder intensity modulator; BC, DC bias controller, EDFA, erbium-doped fibre amplifier; NF, notch filter; DUT, device under test; AOM, acousto-optic frequency shifter; PD, photodetector; RFSA, radio-frequency spectrum analyser.

We examine the inter-modal Brillouin response of this system using nonlinear laser spectroscopy. The stimulated inter-modal Brillouin-scattering spectrum is obtained by measuring phonon-mediated energy transfer between the symmetric and anti-symmetric optical modes of the Brillouin-active waveguide segment. A strong pump wave (*ω*_p_) and weak signal wave (*ω*_s_=*ω*_p_−Ω) enter the symmetric (**E**_1_) and anti-symmetric (**E**_2_) waveguide modes through ports 1 and 2 of mode multiplexer M1, respectively; power guided in these same modes exits ports 1 and 2 of multiplexer M2. As the pump-probe detuning (Ω) is swept through resonance, Brillouin coupling produces energy transfer between pump and probe waves propagating in the symmetric and anti-symmetric modes, yielding the resonant features seen in the gain spectrum (Fig. 3b); throughout this article, we refer to the relative change in the probe intensity as the Brillouin gain.

These Brillouin gain measurements are conducted using the apparatus of Fig. 3a. Both the pump and probe waves are synthesized from the same continuous-wave laser. The laser output (*ω*_p_) is amplified using an erbium-doped fibre amplifier (EDFA) to form the pump wave. A single-frequency probe wave (*ω*_p_−Ω) is synthesized from the same laser output using an electrically driven intensity modulator and an optical notch filter as seen in Fig. 3a. The frequency separation, Ω, between the pump and probe waves is controlled with sub-Hertz precision using a microwave signal generator. Since the probe wave is kept to <200 μW, pump depletion is negligible.

At the device output, the optical waves exiting port 2 of M2 are analysed using wideband heterodyne spectral analysis. As shown in Fig. 3a, signal light exiting port 2 is combined with an optical local oscillator (*ω*_p_+Δ_AOM_) generated by frequency shifting the laser output. As the signal and local oscillator interfere, the intensity of each tone within the signal wave is observed as a unique beat note using a fast photodiode and a microwave spectrum analyser. The Brillouin gain spectrum of Fig. 3b is obtained by measuring the power contained in the output probe wave at frequency *ω*_p_−Ω, as the pump-probe detuning is varied from Ω/2*π*=500 MHz to 9.5 GHz (for more details see Supplementary Note 2).

The frequencies of the identified Brillouin-active phonon modes, labelled B1-9 in Fig. 3b, show good agreement with frequencies predicted through multi-physics simulations (denoted by violet circles along the abscissa). The phononic dispersion curves (computed based on measured device dimensions) for each of the Brillouin-active phonon modes are highlighted in Fig. 3c. Phase-matched coupling occurs where the phonon wavevector (*q*_*m*_(Ω)) of the *m*^th^ Brillouin-active dispersion curve matches the optical wavevector mismatch, Δ*k*(Ω)=*k*_1_(*ω*_p_)−*k*_2_(*ω*_p_−Ω); these frequencies are identified by the intersection between the phonon dispersion curves and the vertical line (gray) indicating computed Δ*k*(Ω)∼4.5 × 10^5^ m^−1^ (for further details on the acoustic modes of this system, see Supplementary Note 7).

The gain spectrum (Fig. 3b) reveals dominant Brillouin resonances, labelled B1, B3 and B6, corresponding to Brillouin-active phonon modes with frequencies of 1.18, 3.09 and 6.03 GHz. Insets within Fig. 3c illustrate the dominant displacement character of each mode. The simulated coupling strengths, obtained through full-vectorial finite element simulations of the type described in ref. 36, produce good agreement with the observed Brillouin nonlinearities for these modes (Table 1). These simulations reveal that the dominant interactions are primarily mediated by photoelasticity (for further details see Supplementary Note 2).

### Dispersive symmetry breaking

Next we examine stimulated inter-modal Brillouin dynamics through both gain and nonlinear power-transfer measurements; throughout these studies, we probe these dynamics using the 6.03 GHz resonance that exhibits the largest Brillouin gain—this resonance is labelled B6 in Fig. 3b. A key characteristic of SIMS produced by coupling to these phonon modes is single-sideband gain. To investigate these dynamics, we perform power-dependent spectral analysis of the probe signal exiting port 2 of M2 as we vary the pump-wave power. Figure 3d shows the spectral content of the transmitted waves obtained through heterodyne measurements when pump and probe light is coupled through the Brillouin-active mode. When the probe detuning is set to the Brillouin resonance frequency and the pump power is varied, we observe single-sideband gain, plotted in the right-most inset of Fig. 3b. Here the measured Stokes (red dots) and anti-Stokes (purple dots) powers are shown as a function of pump power. In contrast to FSBS processes, as the pump power is increased from 0 to 70 mW and the Stokes (red-shifted) sideband experiences gain, no light is scattered to the anti-Stokes order (for further discussion see Supplementary Note 4).

These measurements establish that this inter-modal coupling produces the predicted dispersive symmetry breaking and single-sideband gain necessary to support existing schemes for slow light^27^ and Brillouin-based optical memory^37^. Next, we show that this system produces net optical amplification, necessary to support new laser geometries and robust integrated-photonic performance; a similar figure of merit is required for efficient Brillouin-scattering-induced transparency^7^.

### Single-sideband amplification

Net on-chip optical amplification requires that the Brillouin gain exceeds both linear and nonlinear propagation losses. To quantify the total inter-modal gain and net optical amplification, we perform power-dependent measurements of the gain and loss experienced by the probe wave. Using the experiment diagrammed in Fig. 3a, the pump-probe detuning Ω is swept through the Brillouin frequency Ω_s_ to measure the resonant Brillouin gain spectrum. Three such SIMS gain spectra, for incident on-chip symmetric-mode pump-wave powers of 13, 43 and 88 mW, respectively, are shown in Fig. 3di–iii. These data show a high quality factor (*Q*=Ω_s_/ΔΩ=460) Brillouin resonance at Ω_s_/2*π*=6.03 GHz and 3.5 dB of Brillouin gain at the highest tested pump powers. Peak gain versus pump power, as well as measured linear and nonlinear loss, are plotted in Fig. 3e. Net optical amplification, obtained by subtracting total loss from Brillouin gain, is plotted in Fig. 3f. 2.3 dB of amplification is achieved at the highest tested pump power of 88 mW. These data are used, in conjunction with a model that captures the complete linear and nonlinear waveguide properties, to obtain a Brillouin gain coefficient of *G*_B_=470±30 W^−1^ m^−1^. The measured gain and frequency agree well with simulated values of *G*_B_=430±70 W^−1^m^−1^ and Ω_s_/2*π*=6.07 GHz for this *w*=2.85 μm device.

A variety of inter-modal scattering processes can be realized (beyond that diagrammed in Fig. 1) by addressing the modes of the system in different configurations. For example, by injecting pump light into the anti-symmetric mode, SIMS produces amplification for light in the symmetric mode. Data for four different pump-probe configurations are plotted in Supplementary Note 6.

Significant Brillouin-based optical amplification in silicon relies on low optical propagation losses and large Brillouin gain. In this waveguide system, linear losses for both modes were determined through length-dependent ring-resonator finesse measurements and nonlinear losses were measured through power-dependent intra- and inter-modal transmission measurements (for full details see Supplementary Note 1). Due to reduced spatial overlap between the waveguide modes, nonlinear losses impacting SIMS are 40–70% lower than those for intra-modal scattering, resulting in lower power-dependent losses than those in the FSBS system of ref. 13. This is important as it permits low-threshold amplification and robust operation at high pump powers. Greatly reduced fifth-order losses due to free carrier effects, in particular, are necessary to support high-power laser designs.

### Inter-modal energy transfer

Strong inter-modal Brillouin-coupling permits significant nonlinear power transfer between optical fields and may enable new on-chip optical devices. These dynamics have previously been observed only in highly-nonlinear photonic crystal fibre, where light is scattered between distinct polarization states^28^. This interaction was used as the basis for new forms of active optical isolators^5^. The operation scheme of this active isolator system was made possible through the use of polarization multiplexing which behaves analogously to on-chip mode multiplexing.

In principle, SIMS processes of the type realised here can enable similar forms of nonreciprocal optical devices including active circulators and acousto-optic switches on a silicon chip. To explore the possibility of such operations in an integrated-photonic platform, we measure nonlinear energy transfer in the large-signal regime (that is, where significant energy transfer occurs).

**Figure 4:**
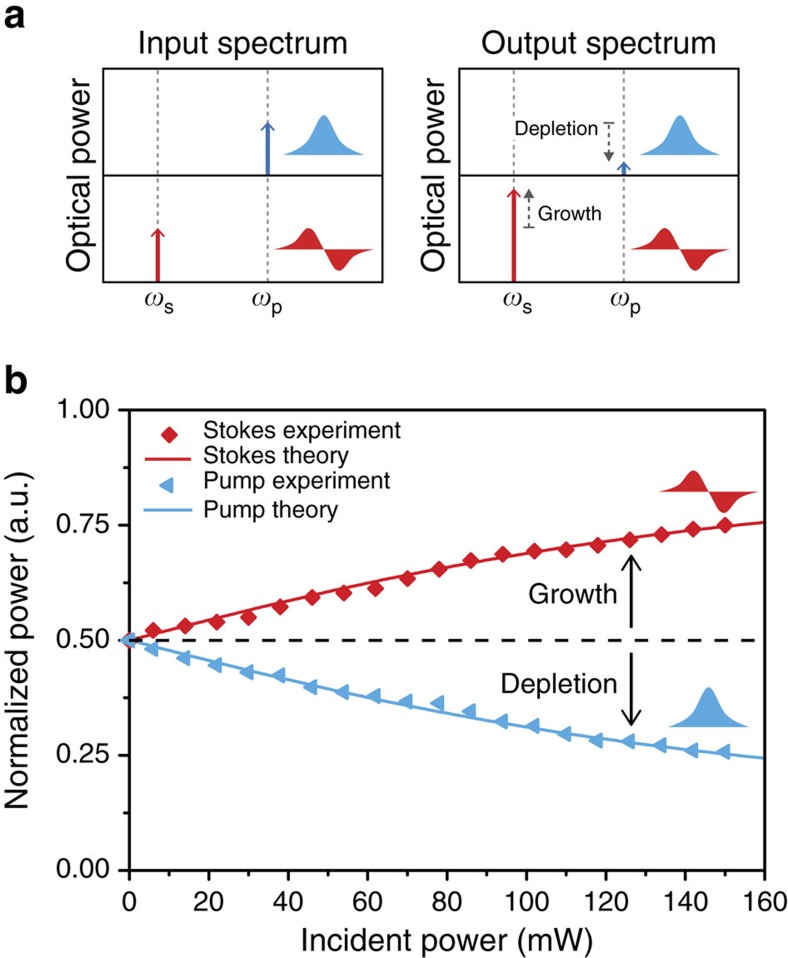
Inter-modal Brillouin energy transfer. (**a**) Diagrams of input and output spectra for the energy transfer experiment. The total change in normalized signal after Brillouin coupling is denoted graphically by dashed arrows in the output spectrum. (**b**) Energy transfer fraction as a function of total incident power for one Brillouin-active device.

We quantify inter-modal Brillouin energy transfer in the large-signal regime using the apparatus of Fig. 3a; here, an additional EDFA (labelled EDFA2) is introduced to boost the probe-wave intensity. Pump and probe waves of equal intensity are injected into modes **E**_1_ and **E**_2_ of the Brillouin-active waveguide, respectively, with pump-probe frequency detuning (Ω) equal to the Brillouin resonance frequency. As the pump and probe waves propagate within the active device region, the pump wave (**E**_1_) is scattered into mode **E**_2_ causing pump depletion and probe-wave gain. As before, pump and probe waves enter and exit the active device region through optical mode multiplexers M1 and M2. Figure 4b plots energy transfer data as a function of combined incident power, with a maximum of 75 mW in both pump and probe waves, for a device with width *w*=2.77 μm and nonlinear coefficient *G*_B_=420±20 W^−1^m^−1^. At the highest tested powers, 50% energy transfer from pump to Stokes is demonstrated.

These results demonstrate a dramatically different regime of operation from the small-signal gain limit. In contrast to the undepleted-pump regime, a significant fraction (many tens of milliwatts) of power is transferred between the participating optical fields. The specific dynamics of this two-wave nonlinear energy transfer process contrast with those of FSBS, where arbitrarily many optical fields participate in the energy transfer process (see Supplementary Note 5). This result also highlights the high-power handling of the unclad silicon membrane waveguide—up to 150 mW powers are guided without adverse effects. The total input power was limited by tight optical confinement in the tapered probe arm of the mode multiplexer, which experiences damage when probe power is in excess of 100 mW. This limit may be relaxed through the use of alternative mode multiplexer designs. Finally, the good agreement achieved between experiment and theory corroborate measurements of nonlinear gain and loss.

The highly-controllable dynamics of SIMS-mediated energy transfer may be applied to create new nonlinear physics on a silicon chip. In addition to new schemes for nonreciprocal devices^5^,^38^, this process supports significant total power transfer even over a short propagation length. ref. 28 demonstrated 97% energy transfer at 300 mW total powers in a 15 m highly-nonlinear photonic crystal fibre with a nonlinear gain coefficient of *G*_B_=1 W^−1^m^−1^. In principle, similar energy transfer efficiencies are possible in an integrated waveguide^32^,^33^. For example, longer propagation lengths (∼7 cm) with similar gain and loss coefficients to the integrated silicon waveguide demonstrated here could permit >90% energy transfer^32^,^33^. However, to reach the theoretical maximum of efficiency possible through this process it will be necessary to further improve the linear and nonlinear optical losses of the waveguide system.

## Discussion

We have shown that this type of on-chip Brillouin scattering produces dispersive symmetry breaking, net optical amplification and appreciable mode conversion. These characteristics permit a variety of processes not previously possible in silicon photonics.

Dispersive symmetry breaking between Stokes and anti-Stokes processes through Brillouin interactions in silicon permits numerous new operations. As discussed above, due to symmetry breaking SIMS supports single-sideband optical amplification and energy transfer. Since Stokes and anti-Stokes scattering are mediated by different phonon modes, several schemes for mode cooling are now possible. In addition to resonator-based cooling schemes^15^, the high nonlinear coupling of this system compares favourably with predictions of necessary gain-power products to observe spontaneous Brillouin cooling in a linear waveguide^16^. The large Brillouin gain and low propagation loss of the membrane waveguide system may also permit the creation of Brillouin-scattering-induced transparency on a silicon chip^7^,^17^. Finally, symmetry breaking enables the adaptation of many traditional technologies based on BSBS, such as existing methods for slow light^27^, beam combining^39^ and optical memory^37^ which are not readily achieved through on-chip FSBS.

The combination of these highly tailorable physics with broadband mode multiplexers^34^,^35^ offers many intriguing possibilities for integrated-photonic systems. In contrast with previous approaches to Brillouin scattering, the operation scheme used for SIMS in silicon eliminates the need for circulators or narrowband filters to separate pump and signal waves—the four-port system discussed here allows automatic multiplexing/demultiplexing of these waves into spatially separate waveguides which can then be routed to other devices on the same chip.

For example, this four-port waveguide system can also be readily adapted into an inter-modal Brillouin laser by connecting ports 2 of M1 and M2 as diagrammed in Fig. 1a. This design creates a cavity for Stokes light while remaining transparent for pump light. This contrasts with traditional Brillouin lasers, where the laser cavity length must be chosen so that the pump and Stokes frequencies precisely align with cavity modes. In particular, this requires that the cavity free spectral range is an integer divisor of the Brillouin frequency shift. SIMS removes this constraint to allow Brillouin lasers with arbitrary cavity lengths on a silicon chip. The gain and power handling demonstrated in the SIMS-active waveguide should be adequate to create such a laser.

Beyond this specific system, SIMS also enables new devices based on inter-modal coupling on a silicon chip. SIMS is a form of active mode conversion, which may have applications in active switching, power routing or on-chip mode-division multiplexing^34^,^35^. The physics of inter-modal Brillouin scattering can be generalized to any number of spatial modes with different symmetries and couplings, in contrast with stimulated inter-polarization scattering. The SIMS-active membrane waveguide also allows geometric tuning of the gain spectrum—the resonant frequency of the Brillouin-active phonons is directly related to the phononic membrane width, unlike in most BSBS systems^10^. (For details see Supplementary Notes 8 and 9). Since the wavevector and frequency of Brillouin-active phonons are highly tailorable, this process can be used as a flexible platform to generate acoustic phonons for on-chip acousto-optic device applications.

In addition, SIMS produces narrow-bandwidth frequency-selective gain, allowing amplification of a specific signal while leaving unwanted side-tones unchanged. This narrowband optical amplification is also important for the creation a narrow-linewidth Brillouin laser. By changing the pump wavelength, the operation wavelength of the Brillouin-active waveguide can be tuned by over 20 nm—this is limited in our experiment by the grating couplers used to couple light onto the chip. A modulated probe will experience uniform amplification, provided that the modulation is within the 13 MHz gain bandwidth. However, once a Brillouin-active phonon is present in the waveguide, it can scatter optical waves (through a linear acousto-optic scattering process) over the optical phase-matching bandwidth of many tens of GHz; this bandwidth scales as 
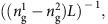
 where 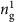 and 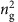 are the group indicies of the two optical modes and *L* is the device length. In this way signal-processing devices based on SIMS can be designed to operate on modulated signals with much greater bandwidth than that of the Brillouin gain. Modulation of the pump allows modulation of the gain experienced by the probe, and has been used to create a fast, nonmagnetic optical switch in photonic crystal fibre^5^.

In conclusion, we have demonstrated stimulated inter-modal Brillouin scattering in an on-chip system. This new nonlinear coupling allows inter-modal amplification and single-sideband energy transfer through highly engineerable control over Brillouin interactions. Through independent photonic and phononic control, we have demonstrated ultralow nonlinear losses intrinsic to the inter-modal coupling and robust acoustic performance. Using this system, we achieved net amplification of 2.3 dB and 50% energy transfer from one optical field to another. This tailorable Brillouin nonlinearity can support a wide range of hybrid photonic-phononic technologies for RF and photonic signal processing, and is readily integrable in silicon photonic systems. This work extends the growing body of research on integrated Brillouin photonics by adding powerful new control over the spatial behaviour and nonlinear dynamics of the Brillouin interaction.

## Methods

### Device fabrication

The silicon waveguides were written on a silicon-on-insulator chip with a 3 μm oxide layer using electron beam lithography on hydrogen silsesquioxane photoresist. Following development, a Cl_2_ reactive ion etch was employed to etch the ridge waveguide structure. After a solvent cleaning step, slots were written to expose the oxide layer, again with electron beam lithography of ZEP520A photoresist and Cl_2_ reactive ion etching. The device was then wet released via a 49% hydrofluoric acid etch of the oxide undercladding. The waveguide structures under test are each comprised of 461 suspended segments.

### Experiment

Both experiments used a pump laser operating around 1,550 nm. For further details on the experimental set-up see Supplementary Note 2.

### Data availability

Data supporting the findings of this study are available upon request.

## Additional information

**How to cite this article:** Kittlaus, E. A. *et al*. On-chip inter-modal Brillouin scattering. *Nat. Commun.* **8,** 15819 doi: 10.1038/ncomms15819 (2017).

**Publisher’s note**: Springer Nature remains neutral with regard to jurisdictional claims in published maps and institutional affiliations.

## Supplementary Material

Supplementary Information Supplementary Figures, Supplementary Table, Supplementary Notes and Supplementary References

## Figures and Tables

**Table 1:** Comparison of measured and simulated Brillouin gain.

**Mode label**	**Simulated** *G*_B_	**Measured** *G*_B_
B1	<40 W^−1^ m^−1^	30±5 W^−1^ m^−1^
B3	155±30 W^−1^ m^−1^	140±20 W^−1^ m^−1^
B6	430±70 W^−1^ m^−1^	470±30 W^−1^ m^−1^
