# Supplementary material for: On-chip inter-modal Brillouin scattering
Source: Nat Commun. 2017 Jul 7;8:15819. doi: 10.1038/ncomms15819 (PMC5504300; doi:10.1038/ncomms15819)
Supplement: Supplementary Information — Supplementary Figures, Supplementary Table, Supplementary Notes and Supplementary References [file ncomms15819-s1.pdf]

Supplementary Note 1. Nonlinear Coefficients and Loss Model

We calculate the effective guided-wave nonlinear coefficients using an electromagnetic-wave finite-element model (FEM) simulation. These nonlinear coefficients are enhanced compared to their bulk values due to the nontrivial vectorial nature of the highly-confined optical modes [1]. Using the method of Ref. [1] and a bulk value of $n_2 = 4.5 \times 10^{-18}$ m^2W^{-1} [2], we calculate guided wave Kerr coefficients of $\gamma_{\mathrm{k}}^{11} = 74 \pm 11$ $m^{-1}W^{-1}$, $\gamma_{\mathrm{k}}^{22} = 71 \pm 11$ $m^{-1}W^{-1}$, and $\gamma_{\mathrm{k}}^{12} = \gamma_{\mathrm{k}}^{21} = 44 \pm 7$ $m^{-1}W^{-1}$, where the superscripts to γ^{ij} identify the modes participating in the nonlinear coupling. Here, superscript 1 (2) identifies participation of the fundamental (first excited) waveguide mode. Using a bulk value for the two-photon coefficient of $\beta_{\mathrm{TPA}} = 7.9 \times 10^{-12}$ mW^{-1} we find $\beta^{11} = 34 \pm 10$ $m^{-1}W^{-1}$, $\beta^{22} = 30 \pm 9$ $m^{-1}W^{-1}$, and $\beta^{12} = \beta^{21} = 20 \pm 6$ $m^{-1}W^{-1}$. Here we use units of guided-wave power, i.e. $\gamma_{\mathrm{k}} \approx n_2\omega/(c \cdot A_{\mathrm{eff}})$ and $\beta \approx \beta_{\mathrm{TPA}}/A_{\mathrm{eff}}$, where A_{eff} is the effective nonlinear mode area.

We analyze the linear and nonlinear losses of our waveguide system through measurements of both intra- and inter-modal nonlinear loss for each mode. We then use models of the device physics to extract the nonlinear loss coefficients of our waveguides from these data. To an excellent approximation, the intra-modal propagation loss in a single-mode silicon waveguide system is captured by the differential equation

$$\frac{dP_i}{dz} = -\alpha^i P_i - \beta^{ii} P_i^{\ 2} - \gamma^{iii} P_i^{\ 3} \,. \tag{1}$$

Here α^i is the linear loss coefficient for the ith waveguide mode and γ^{iii} denotes the intra-modal nonlinear loss coefficient due to TPA-induced free carrier absorption (FCA). Fitting measured nonlinear transmission measurements (Supplementary Fig. 1a,c) to this model allows the measurement of γ^{iii}.

In the presence of a bright pump P_i, the inter-modal nonlinear loss experienced by a weak field P_j in a different mode is given by the additional equation

$$\frac{dP_j}{dz} = -\alpha^j P_j - 2\beta^{ji} P_i P_j - \gamma^{jii} {P_i}^2 P_j \,. \quad (2)$$

comparing this equation to measured cross-modal nonlinear loss with the calculated value for β^{ji} allows determination of γ^{jii}.

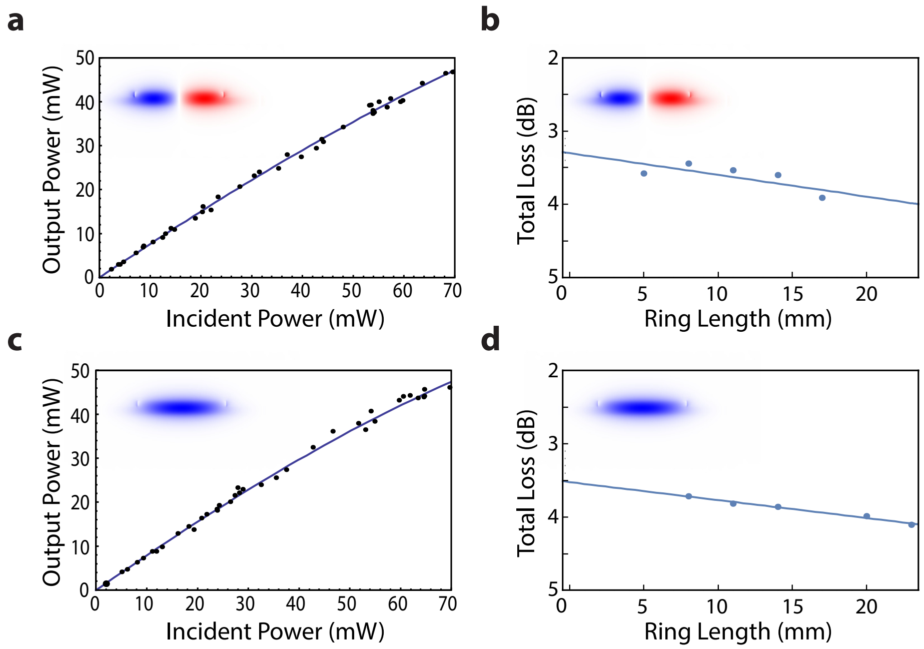

Supplementary Figure 1. Linear and nonlinear loss measurements at $\lambda \approx 1550$ nm for the membrane waveguide. Panels (a) and (b) plot input vs. output power for the device under study and loss vs. ring resonator length for the anti-symmetric mode $\boldsymbol{E}_2$. (c) and (d) plot these data for the fundamental mode $\boldsymbol{E}_1$.

Linear loss α^i is determined by measuring the intrinsic *Q*-factor of ring resonators fabricated from multimode silicon waveguides of different lengths. The results of these studies (Plotted in Supplementary Figure 1b,d) determine linear propagation losses to be 0.24 ± 0.02 dB/cm ($\alpha^1 = 5.5 \pm .5$ m^{-1}) for the fundamental mode and 0.30 ± 0.12 dB/cm ($\alpha^2 = 6.8 \pm 2.7$ m^{-1}) for the higher-order mode. To measure free carrier losses, power-dependent transmission data were fit to the nonlinear loss models using Mathematica and assuming the values of β^{11}, β^{12}, and β^{22} calculated in Supplementary Note 1. The intra-modal data plotted in Supplementary Figure 1c-d are best fit with $\gamma^{111} = 900 \pm 400$ m^{-1}W^{-2} and $\gamma^{222} = 720 \pm 430$ m^{-1}W^{-2}. These data are consistent with a free carrier lifetime of 1.2 ns according to a Drude-Sommerfeld model of free carrier absorption [3] or a lifetime of 1.5 ns according to a Drude-Lorentz model [4, 5]. According to the overlap integral method of Ref. [3], γ^{211} is expected to be 41% of γ^{111} in magnitude.

Measurements of small signal cross-modal nonlinear loss of .45 dB at 76 mW pump powers are consistent with $\gamma^{211} \approx \gamma^{122}$ = 310 ± 200 $m^{-1}W^{-2}$, which is around 34% of γ^{111}, consistent with this calculation.

Supplementary Note 2. Brillouin Gain Model and Measurement

In the Brillouin gain experiment, a strong pump field is used to amplify a weak Stokes signal. In the small signal limit ($P_{\mathrm{p}} \gg P_{\mathrm{s}}$), the coupled differential equations relating the propagating powers in the two fields in the presence of nonlinear loss are [3]:

$$\frac{1}{P_{\mathrm{p}}(z)}\frac{dP_{\mathrm{p}}(z)}{dz} = -\left(\alpha^{\mathrm{p}} + \beta^{\mathrm{pp}}P_{\mathrm{p}}(z) + \gamma^{\mathrm{ppp}}P_{\mathrm{p}}^{2}(z)\right), \tag{3}$$

$$\frac{1}{P_{\mathrm{s}}(z)}\frac{dP_{\mathrm{s}}(z)}{dz} = -\alpha^{\mathrm{s}} + \left(G_{\mathrm{B}}(\Omega) - 2\beta^{\mathrm{sp}} - \gamma^{\mathrm{spp}}P_{\mathrm{p}}(z)\right)P_{\mathrm{p}}(z). \tag{4}$$

Here α^{i}, β^{ij} and γ^{ijk} are the loss coefficients discussed in the previous section with the superscripts p and s referring to the indices of the pump and Stokes modes. $P_{\mathrm{p}}(z)$ and $P_{\mathrm{s}}(z)$ are the pump and Stokes powers at position z along the device length, and $\mathrm{G_B}(\Omega)$ is the inter-modal Brillouin gain coefficient. The device acts as a linear amplifier for the Stokes signal, with total amplification dependent on the input pump power.

After passing through the Brillouin-active waveguide segment and mode de-multiplexer, probe light is combined with a frequency shifted version of the incident pump-wave (ω_{p}), which serves as an optical local oscillator (LO) as the basis for heterodyne signal analysis. The pump wave is shifted to frequency $\omega_{\mathrm{LO}} = \omega_{\mathrm{p}} + \Delta_{\mathrm{AOM}}$ using a fiber-coupled acoustic-optic modulator which produces a frequency shift of $\Delta_{\mathrm{AOM}} =$ 44 MHz. Throughout heterodyne measurements, the local-oscillator power P_{LO} and incident signal power P_{s}^{0} are held constant. As the pump-probe detuning is swept through the Brillouin resonance, the probe power experiences a frequency dependent change:

$$P_{\mathrm{s}} = P_{\mathrm{s}}^{0} + \Delta P_{\mathrm{s}}(\Omega). \tag{5}$$

The power in the probe field is measured via the power of the RF beat-note at frequency $\Omega + \Delta_{\mathrm{AOM}}$ between probe and reference fields ($\propto P_{\mathrm{s}}P_{\mathrm{LO}}$) incident on a fast photodetector. This resulting RF power is:

$$P_{\mathrm{RF}}(\Omega + \Delta_{\mathrm{AOM}}) = \eta P_{\mathrm{LO}}\left(P_{\mathrm{s}}^{0} + \Delta P_{\mathrm{s}}(\Omega)\right). \tag{6}$$

Here η is a coefficient of proportionality related to the detector efficiency. To determine the change in probe power, this signal is normalized to the RF power in the absence of Brillouin coupling:

$$\frac{P_{\mathrm{RF}}(\Omega+\Delta_{\mathrm{AOM}})}{\eta P_{\mathrm{LO}} P_{\mathrm{s}}^{0}} = 1 + \frac{\Delta P_{\mathrm{s}}(\Omega)}{P_{\mathrm{s}}^{0}}. \tag{7}$$

This is the normalized change in probe signal power after Brillouin coupling.

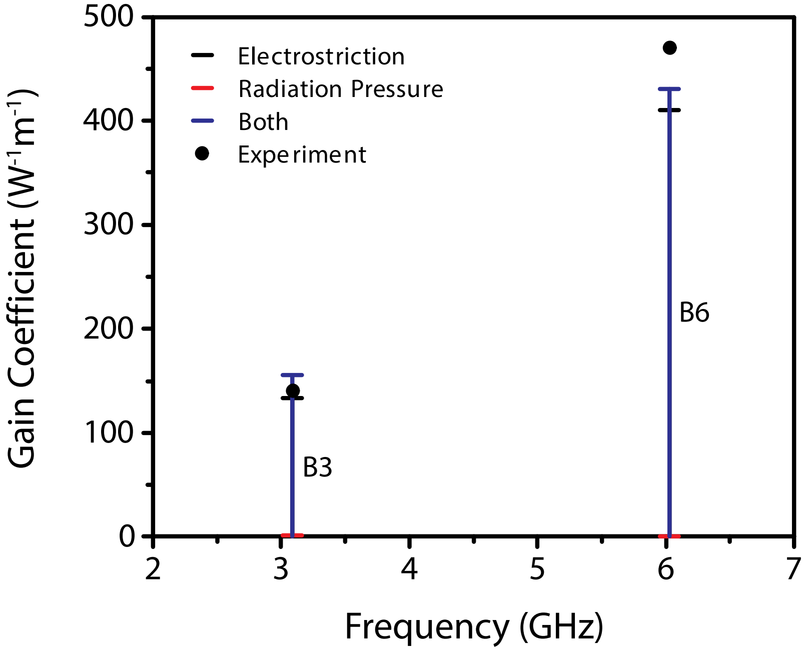

Supplementary Figure 2. Simulated gain coefficients for the dominant Brillouin-active modes B3 and B6 including contributions from electrostriction (black bar), radiation pressure (red bar), and the combined effect (blue bar). Experimental data is plotted as black dots. The margin of error of the simulations is estimated to be ±15% due to uncertainties in material parameters and device structure.

The measured data from the Brillouin gain experiment are fit to this model to determine the gain coefficient for the dominant Brillouin-active mode B6 of $G_B = 470 \pm 30$ $m^{-1}W^{-1}$. The Brillouin gain coefficient is numerically simulated to be $G_B = 430 \pm 70$ $m^{-1}W^{-1}$ through a full-vectorial simulation with the commercially available finite-element method software COMSOL according to the method Ref. [6], with values for the photoelastic tensor components for silicon $(p_{11},p_{12},p_{44}) = (-0.09,0.017,-0.051)$ [7]. As depicted in Fig. 2 of the main article, the Brillouin coupling is mediated primarily by the *x*-component of the electrostrictive force. This is plotted in Supplementary Figure 2 for the two modes B3 and B6 which experience the strongest Brillouin gain. The measured and simulated values of the Brillouin gain for mode B3 are $G_B = 140 \pm 20$ m-

$^{1}W^{-1}$ and $G_B = 155 \pm 30$ $m^{-1}W^{-1}$, respectively. The remaining modes with measured $G_B \leq 40 \pm$ $m^{-1}W^{-1}$ have simulated values in this range but are difficult to calculate precisely due to uncertainties in the values of and contributions from the various photoelastic tensor components. For example, mode B1 with measured gain coefficient $G_B = 30 \pm 5$ $m^{-1}W^{-1}$ when numerically simulated has contributions from each of (p_{11}, p_{12}, p_{44}) that are similar in magnitude but differing in sign.

Supplementary Note 3. Mode Multiplexers

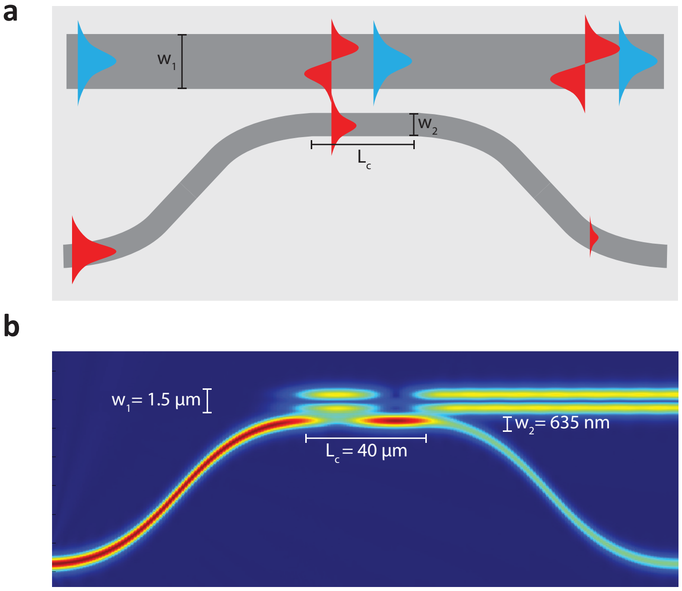

Supplementary Figure 3. (a) Schematic of the mode selective directional coupler. (b) Simulation of coupling into the first excited mode with typical dimensions. In this particular simulation, 78% of the light is converted into the first excited spatial mode.

We employ mode-selective directional couplers to multiplex light into two distinct spatial modes of the hybrid photonic-phononic waveguide [8]. Before the mode multiplexer, pump and Stokes light are spatially separated, each guided by a single-mode 450 nm wide ridge waveguide. The waveguide guiding the pump field is tapered to a width of 1.5 µm which supports multimode operation. Simultaneously, the waveguide guiding the probe field is tapered to a width (635 nm) such that its effective index closely matches that of the first excited mode of the 1.5 µm waveguide. As depicted in Supplementary Fig. 3, the narrow waveguide then channels the Stokes wave towards the multimode waveguide to permit evanescent coupling into the higher order

mode. From a coupled mode theory formulation [9], one can show that the fraction of light coupled (C) into the higher order mode is

$$C = \frac{\kappa_{12}}{\beta_0}\sin(\beta_0), \quad (8)$$

$$\beta_0 = \sqrt{\kappa_{21}\kappa_{12} + \frac{(\beta_1 - \beta_2)^2}{2}}. \quad (9)$$

Here κ_{12} and κ_{21} are the coupling coefficients and β_1 and β_2 are the propagations constants of the modes of interest. The above equation shows that near unity power transfer is possible given identical effective indices. It also highlights that both the magnitude and phase of the coupling change dramatically with effective index mismatch, permitting strong coupling into the desired mode with little crosstalk to other spatial modes.

Using the process outlined in the methods section, we fabricate the mode multiplexers on a silicon on insulator platform. These directional couplers achieve 60 to 95 percent coupling from the fundamental mode of the 635 nm waveguide to the first excited mode of the multimode waveguide depending on fabrication parameters, with a typical value of around 75%.

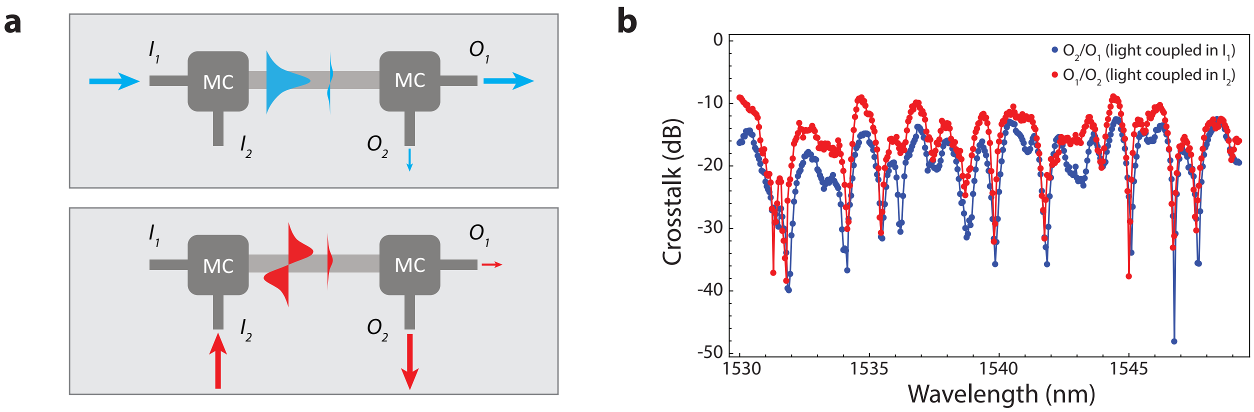

Supplementary Figure 4. (a) Schematic for crosstalk measurement: Light injected into input port (I-1) is coupled into the fundamental mode with a small amount of light in the first excited mode. Light coupled into input port (I-2) is converted into the first excited mode with some residual light in the fundamental. (b) Typical crosstalk of a multiplexer and demultiplexer in series. Crosstalk is calculated by dividing powers of the light from the two output ports (O-1 and O-2).

After passing through the active device region, an identical coupler de-multiplexes the two modes into two spatially separated single-mode waveguides. Any residual light (light not demultiplexed) in the first excited mode of the main waveguide is removed by a spatial mode

filter at the output. Ideally, each port of the multiplexer couples uniquely to its respective mode. In practice, some small amount of light is (de)multiplexed into the incorrect mode or port. Supplementary Figure 4 shows a schematic for the crosstalk measurement and the crosstalk for a typical mode multiplexer and de-multiplexer in series. Median crosstalk values for this configuration are typically -15 to -20 dB, though all experiments are carried out in regions with total crosstalk less than -20 dB.

Supplementary Note 4. SIMS vs. FSBS Dynamics

In the case of forward intra-modal SBS, the same phonon is phase-matched to both Stokes and anti-Stokes processes. As a result, the dynamics of these fields are coupled, and the equations of motion for the participating optical and acoustic envelope fields in a waveguide can be written as follows [10]:

$$\frac{\partial \bar{B}}{\partial t} + v_0 \frac{\partial \bar{B}}{\partial z} = i(\Omega - \Omega_{\mathrm{B}})\bar{B} - i\left(g_0^* \bar{A}_{\mathrm{s}}^* \bar{A}_{\mathrm{p}} + g_1^* \bar{A}_{\mathrm{p}}^* \bar{A}_{\mathrm{as}}\right), \tag{10}$$

$$\frac{\partial \bar{A}_{\mathrm{p}}}{\partial t} + v_{\mathrm{p}} \frac{\partial \bar{A}_p}{\partial z} = -i(g_0 \bar{A}_{\mathrm{s}} \bar{B} + g_1^* \bar{B}^* \bar{A}_{\mathrm{as}}), \tag{11}$$

$$\frac{\partial \bar{A}_{\mathrm{s}}}{\partial t} + v_{\mathrm{s}} \frac{\partial \bar{A}_{\mathrm{s}}}{\partial z} = -i g_0^* \bar{B}^* \bar{A}_{\mathrm{p}}, \tag{12}$$

$$\frac{\partial \bar{A}_{\mathrm{as}}}{\partial t} + v_{\mathrm{as}} \frac{\partial \bar{A}_{as}}{\partial z} = -i g_1 \bar{A}_{\mathrm{p}} \bar{B}. \tag{13}$$

Here $\bar{B}$, $\bar{A}_{\mathrm{p}}$, $\bar{A}_{\mathrm{s}}$, and $\bar{A}_{\mathrm{as}}$ are the envelope functions for the phonon field and pump, Stokes, and anti-Stokes fields, respectively. v_0, v_{p}, v_{s}, and v_{as} are the corresponding group velocities, $\Omega = \omega_{\mathrm{p}} - \omega_{\mathrm{s}}$ is the pump/Stokes detuning and Ω_{B} is the Brillouin resonance frequency, and g_0 and g_1 are the Brillouin coupling strengths for Stokes and anti-Stokes processes, respectively, and are assumed to be equal here. Since Stokes and anti-Stokes processes couple to the pump through the same phonon field, appreciable light is scattered from the pump to both fields.

By contrast, through SIMS Stokes and anti-Stokes processes couple through phonons with distinct wavevector. As a result, the two processes are de-coupled, and the equations of motions for the envelope functions can be written as follows [10]:

$$\frac{\partial \bar{B}_{\mathrm{s}}}{\partial t} + v_0 \frac{\partial \bar{B}_{\mathrm{s}}}{\partial z} = i(\Omega - \Omega_{\mathrm{s}})\bar{B}_{\mathrm{s}} - i g_0^* \bar{A}_{\mathrm{s}}^* \bar{A}_{\mathrm{p}}, \tag{14}$$

$$\frac{\partial \bar{A}_{\mathrm{p}}}{\partial t} + v_{\mathrm{p}} \frac{\partial \bar{A}_{\mathrm{p}}}{\partial z} = -i g_0 \bar{A}_{\mathrm{s}} \bar{B}_{\mathrm{s}}, \tag{15}$$

$$\frac{\partial \bar{A}_{\mathrm{s}}}{\partial t} + v_{\mathrm{s}} \frac{\partial \bar{A}_{\mathrm{s}}}{\partial z} = -i g_0^* \bar{B}_{\mathrm{s}}^* \bar{A}_{\mathrm{p}}. \tag{16}$$

A similar set of equations are found for anti-Stokes scattering:

$$\frac{\partial \bar{B}_{\mathrm{as}}}{\partial t} + v_0 \frac{\partial \bar{B}_{\mathrm{as}}}{\partial z} = i(\Omega - \Omega_{\mathrm{as}})\bar{B}_{\mathrm{as}} - i g_1^* \bar{A}_{\mathrm{p}}^* \bar{A}_{\mathrm{as}}, \tag{17}$$

$$\frac{\partial \bar{A}_{\mathrm{p}}}{\partial t} + v_{\mathrm{p}} \frac{\partial \bar{A}_{\mathrm{p}}}{\partial z} = -i g_1^* \bar{B}^* \bar{A}_{\mathrm{as}}, \tag{18}$$

$$\frac{\partial \bar{A}_{\mathrm{as}}}{\partial t} + v_{\mathrm{as}} \frac{\partial \bar{A}_{\mathrm{as}}}{\partial z} = -i g_1 \bar{A}_{\mathrm{p}} \bar{B}. \tag{19}$$

Note that the phonon mediating the anti-Stokes process, $\bar{B}_{\mathrm{as}}$, is different than the phonon $\bar{B}_{\mathrm{s}}$ mediating Stokes coupling. These equations produce the single-sideband optical coupling inherent to SIMS.

Supplementary Note 5. Energy Transfer model

In the case where the pump and Stokes waves are similar in magnitude, additional nonlinear terms must be added to the SIMS power evolution equations [3]:

$$\frac{1}{P_{\mathrm{p}}(z)} \frac{dP_{\mathrm{p}}(z)}{dz} = -\left(\alpha^{\mathrm{p}} + \beta^{\mathrm{pp}} P_{\mathrm{p}}(z) + \gamma^{\mathrm{ppp}} P_{\mathrm{p}}^2(z)\right) - \left(2\beta^{\mathrm{ps}} - G_{\mathrm{B}}(\Omega) + 4\gamma^{\mathrm{pps}} P_{\mathrm{p}}(z) + \gamma^{\mathrm{pss}} P_{\mathrm{s}}(z)\right) P_{\mathrm{s}}(z), \tag{17}$$

$$\frac{1}{P_{\mathrm{s}}(z)} \frac{dP_{\mathrm{s}}(z)}{dz} = -\left(\alpha^{\mathrm{s}} + \beta^{\mathrm{ss}} P_{\mathrm{s}}(z) + \gamma^{\mathrm{sss}} P_{\mathrm{s}}^2(z)\right) - \left(2\beta^{\mathrm{sp}} + G_{\mathrm{B}}(\Omega) + 4\gamma^{\mathrm{ssp}} P_{\mathrm{s}}(z) + \gamma^{\mathrm{spp}} P_{\mathrm{p}}(z)\right) P_{\mathrm{p}}(z). \tag{18}$$

Multiple strong waves present in a nonlinear waveguide experience additional loss due to cross-field losses. This effect hinders energy transfer efficiency at high powers, suggesting that longer devices operating at lower powers or larger effective mode areas are amenable to highly-efficient nonlinear energy transfer.

It is worth noting that the coupling for this form of nonlinear energy transfer involves only two participating fields. This contrasts with intra-modal FSBS, where strong coupling results in

stimulated energy transfer to multiple successive orders. Data for different input powers is plotted for the present work (SIMS) and for intra-modal FSBS in Supplementary Figure 5.a-b.

Energy transfer through traditional FSBS energy is intrinsically limited to approximately 50% due to energy transfer to higher order comb lines [11, 12]. This effect results from the FSBS-active phonon phase-matching to arbitrarily many Brillouin processes. The phonon that mediates SIMS phase matches unidirectionally from one optical mode to another, precluding any cascading effects and permitting in principle complete energy transfer from one field to another.

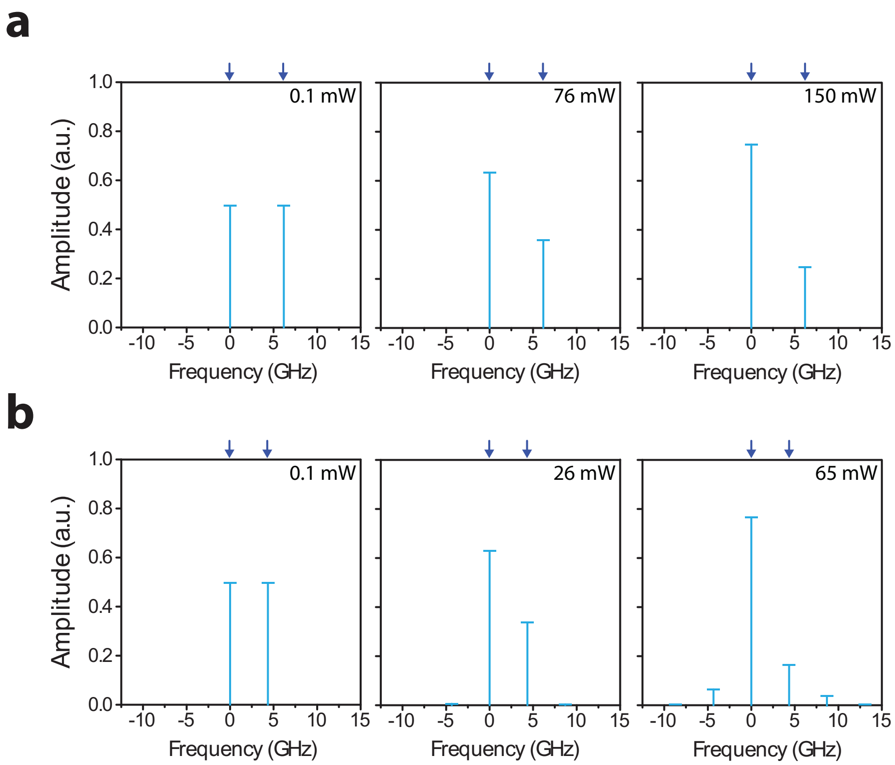

Supplementary Figure 5. Experimental data at different total incident powers showing nonlinear energy transfer for SIMS (a) and FSBS (b). The FSBS data are from Ref. [12]. The blue arrows above each panel denote where two equal-intensity drive tones are injected into the waveguide. While SIMS only couples light between two optical fields, at high powers FSBS produces cascaded energy transfer.

Supplementary Note 6. Red- vs. Blue-shifted probe data

The gain data in the main text are for a signal wave guided in the first-excited optical mode of a ridge waveguide while pump light is guided in the fundamental mode. The opposite configuration is also possible and supports comparable amplification. Conversely, a small signal that is blueshifted from a strong pump by the Brillouin resonance frequency experiences attenuation—this is no different than the strong pump depletion limit, or the near-unity power transfer limit of the energy transfer experiment from the main article. Supplementary Figure 6 plots data for these four pump/probe configurations in a Brillouin-active waveguide with w = 2.77 μm, $G_B = 404 \pm 20\ m^{-1}W^{-1}$, and $\Omega_B/2\pi$ = 6.16 GHz. This experiment used asymmetric Y-junction spatial mode converters which allow narrowband near-unity coupling into each optical mode [13].

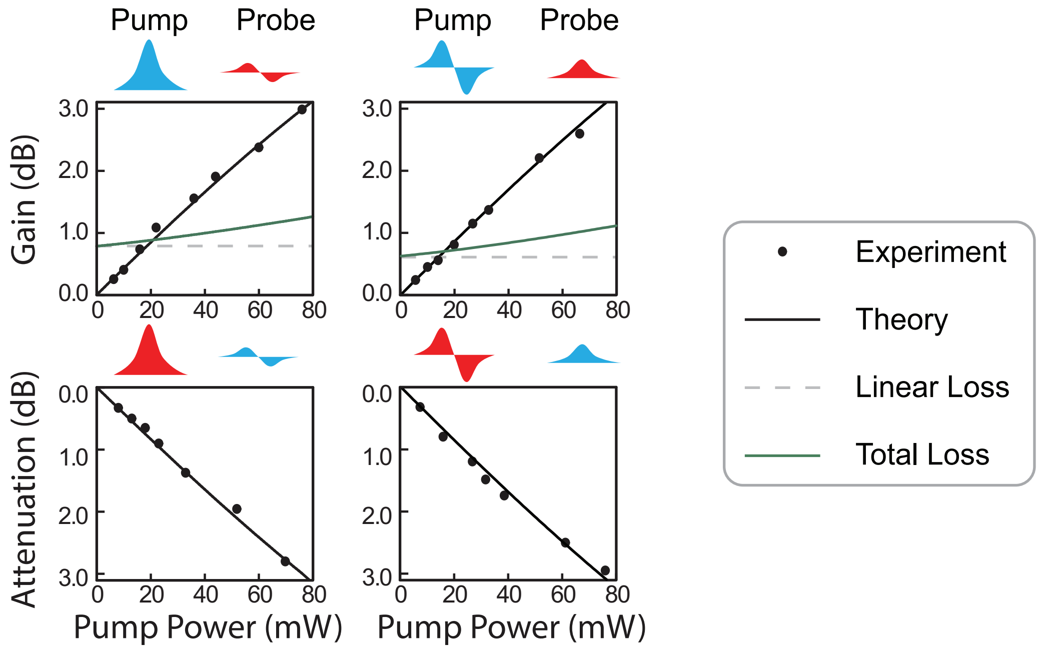

Supplementary Figure 6. Plots of probe gain or attenuation for a probe signal in four-different pump-probe configurations. The top left panel corresponds to the pump/probe configuration presented in the main article. The top two panels show gain for a weak probe red-shifted from the pump by the Brillouin resonance frequency, while the bottom two panels show attenuation of a weak probe which is blue-shifted from the pump by the Brillouin resonance frequency.

Supplementary Note 7. Elastic Mode Simulations

Fig. 2 in the main text depicts acoustic dispersion for the Brillouin-active modes observed. There are many other resonant modes of the membrane waveguide system which do not couple appreciably through SIMS. Complete acoustic dispersion relations for frequencies $\Omega/2\pi < 10$ GHz and wavevector $q < 10^6$ m^{-1} are plotted in Supplementary Figure 7.

The Lamb wave-like elastic modes of the system can be separated into four categories based on their direction of motion. The first type is modes experiencing compression and rarefaction in the x-direction (red lines in Supplementary Fig. 7). The 3 and 6 GHz modes which couple most strongly to the optical waveguide modes through SIMS are of this type. The displacement field of an example mode at around 4.3 GHz is plotted in Supplementary Figure 7.i.

The second family of modes are asymmetric Lamb wave-like modes with in-plane motion in the x- and y-directions, as shown by the 3.5 GHz mode plotted in Supplementary Figure 7.ii. These elastic modes are similar to transverse acoustic waves on a string or thin membrane. The third family of modes experiences compressive displacement in the longitudinal (z) direction. An example mode at 4.7 GHz is plotted in in Supplementary Figure 7.iii. Finally, three modes with linear dispersion correspond to the fundamental transverse x- and y-displacement modes and the z-compressive mode.

These modes are simulated using a full-vectorial elastic wave method using the commercially-available finite element model package COMSOL.

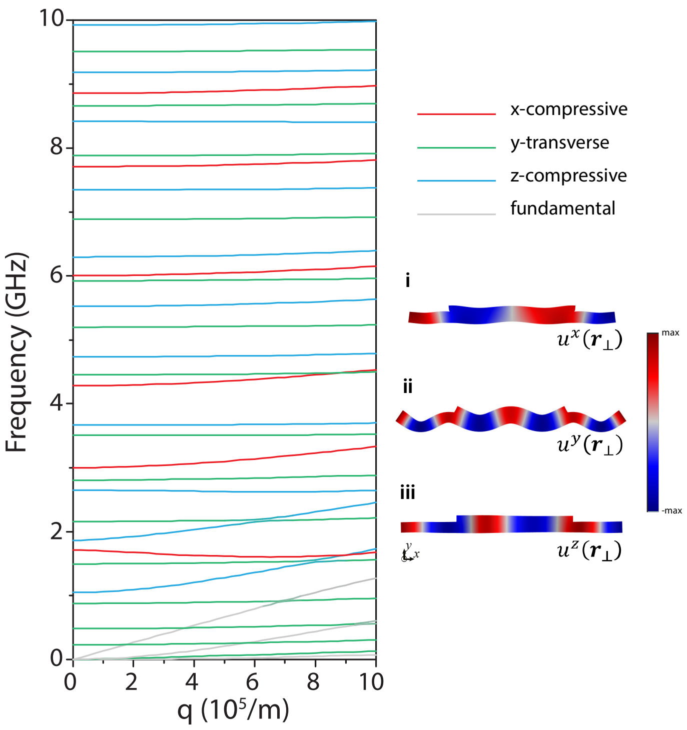

Supplementary Figure 7. Acoustic dispersion relations for GHz-frequency guided acoustic modes of the suspended membrane waveguide. The modes are categorized according to their displacement field and examples of three types are plotted in panels i-iii.

Supplementary Note 8. Brillouin Resonance Frequency Tuning vs. Device Width

The frequencies of the guided acoustic waves in the Brillouin-active membrane waveguide are directly related to device dimension. By lithographically varying the dimensions of the system, this allows frequency tuning of the Brillouin gain spectrum. Supplementary Figure 8 depicts theory and data for the frequencies of the two strongest Brillouin-active resonances around 3 and 6 GHz for a range of device widths. The predicted frequencies are calculated using COMSOL as in the previous section. Good agreement is achieved between experiment and theory over the tested range of dimensions.

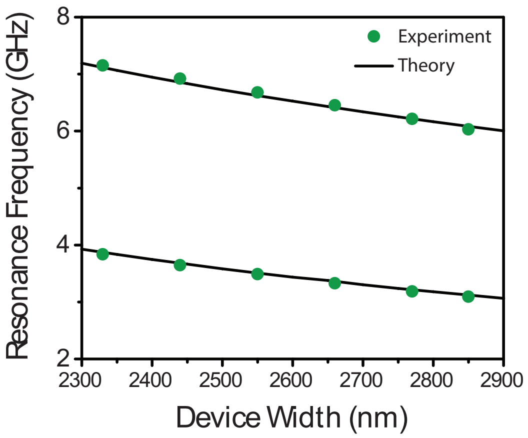

Supplementary Figure 8. Simulated and measured acoustic frequencies for the two acoustic modes with the largest Brillouin gain as a function of device width. Good agreement is achieved between experiment and theory.

Supplementary Note 9. Sensitivity to Dimensional Broadening

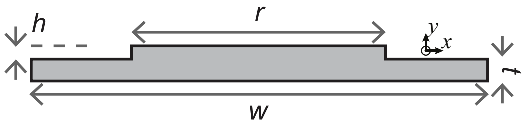

Supplementary Figure 9. Device cross-section labeling the various dimensions which specify the device design.

As has been previously studied, the suspended ridge waveguide design supports a relatively high tolerance to dimensionally-induced broadening of the Brillouin resonance lineshape. In contrast to traditional forward-SBS, SIMS is significantly more sensitive to variations in device thickness and ridge waveguide height due to an additional dispersion-related broadening mechanism: As studied in Ref. [14], there are two dominant sources of inhomogeneous broadening of the gain spectrum in nanoscale Brillouin-active waveguides. The first is direct broadening via acoustic frequency shifting of the resonance as device dimensions vary along the propagation length. The second results from variations of the optical wavenumber that cause frequency shifts via the phase matching condition. Traditional FSBS, which couples to a phonon with vanishingly small wavevector, is devoid of the second broadening mechanism. Since the SIMS process studied here has a nonvanishing wavevector, this additional issue could lead to a degradation in measured

effective quality factor. However, the acoustic quality factors for the SIMS phonon mode compare well with existing FSBS systems, boding well for the potential of further system scaling.

Simulated sensitivities in resonance frequency of the 6 GHz acoustic mode for small changes in device dimension (labeled in Supplementary Figure 9) are tabulated in Supplementary Table 1. These calculated values take into account both potential broadening mechanisms.

Supplementary Table 1

**Dimension**	**Description**	**Sensitivity to Dimension Error**
w	Total membrane width	1.7×10^{6} Hz/nm
t	Membrane Thickness	1.7×10^{6} Hz/nm
r	Ridge waveguide width	9.2×10^{5} Hz/nm
h	Ridge waveguide height	1.4×10^{6} Hz/nm
	Ridge offset from center	$<<10^{5}$ Hz/nm

Supplementary Table 1. Simulated SIMS-active phonon frequency changes with variations in device dimensions. Changes in device dimension along its length can lead to Brillouin resonance broadening.

Supplementary References

[1] S. Afshar V and T. M. Monro, Opt. Express **17**, 2298-2318 (2009).

[2] M. Dinu, F. Quochi, and H. Garcia, Appl. Phys. Lett. **82**, 2954-2956 (2003).

[3] C. Wolff, P. Gutsche, M. J. Steel, B. J. Eggleton, and C. G. Poulton, J. Opt. Soc. Am. B **32**, 1968-1978 (2015).

[4] R. Dekker, N. Usechak, M. Först, and A. Driessen, J. Phys. D **40**, R249-R271 (2007).

[5] R. Claps, V. Raghunathan, D. Dimitropoulos, and B. Jalali, Opt. Express **12**, 2774-2780 (2004).

[6] W. Qiu, P. T. Rakich, H. Shin, H. Dong, M. Soljačić, and Z. Wang, Opt. Express **21**, 31402-31419 (2013).

[7] D. K. Biegelsen, Phys. Rev. Lett. **32**, 1196-1199 (1974).

[8] D. Dai, J. Wang, & Y. Shi. *Opt. Lett. 38*, 1422-1424 (2013).

[9] L. Luo, N. Ophir, C. Chen, L.H. Gabrielli, C.B. Poitras, K. Bergmen, and M. Lipson. Nat. Commun. **5**, 3069 (2014).

[10] P. Kharel, R.O. Behunin, W.H. Renninger, and P.T. Rakich. *Phys. Rev. A* **93**, 063806 (2016).

[11] M.S. Kang, A. Nazarkin, A. Brenn, and P.StJ. Russell. *Nat. Phys.* **5**, 276-280 (2009).

[12] E.A. Kittlaus, H. Shin, and P.T. Rakich. *Nat. Photon.* **10**, 463–467 (2016).

[13] J.B. Driscoll, R.R. Grote, B. Souhan, J.I. Dadap, M. Lu, and R.M. Osgood. *Opt. Lett.* **38** 1854-1856 (2013).

[14] C. Wolff, R. Van Laer, M.J. Steel, B.J. Eggleton, and C.G. Poulton. *New. J. Phys.* **18** 025006 (2016).
